# Clinical characteristics and neurodevelopmental outcomes of children with tuberculous meningitis and hydrocephalus

**DOI:** 10.1111/dmcn.13054

**Published:** 2016-02-16

**Authors:** Ursula K Rohlwink, Kirsty Donald, Bronwyn Gavine, Llewellyn Padayachy, Jo M Wilmshurst, Graham A Fieggen, Anthony A Figaji

**Affiliations:** ^1^ Division of NeurosurgeryPaediatric NeurosurgeryRed Cross War Memorial Children's HospitalUniversity of Cape TownCape TownSouth Africa; ^2^ Clinical and Infectious Disease Research InitiativeInstitute of Infectious Disease and Molecular MedicineUniversity of Cape TownCape TownSouth Africa; ^3^ Division of Developmental PaediatricsDepartment of Paediatrics and Child HealthRed Cross War Memorial Children's HospitalUniversity of Cape TownCape TownSouth Africa; ^4^ Faculty of Health SciencesUniversity of Cape TownCape TownSouth Africa; ^5^ Paediatric NeurologyDepartment of Paediatrics and Child HealthRed Cross War Memorial Children's HospitalUniversity of Cape TownCape TownSouth Africa

## Abstract

**Aim:**

Tuberculous meningitis (TBM) is a lethal and commonly occurring form of extra‐pulmonary tuberculosis in children, often complicated by hydrocephalus which worsens outcome. Despite high mortality and morbidity, little data on the impact on neurodevelopment exists. We examined the clinical characteristics, and clinical and neurodevelopmental outcomes of TBM and hydrocephalus.

**Method:**

Demographic and clinical data (laboratory and radiological findings) were prospectively collected on children treated for probable and definite TBM with hydrocephalus. At 6 months, clinical outcome was assessed using the Paediatric Cerebral Performance Category Scale and neurodevelopmental outcome was assessed with the Griffiths Mental Development Scale – Extended Version.

**Results:**

Forty‐four patients (median age 3y 3mo, range 3mo–13y 1mo, [SD 3y 5mo]) were enrolled. The mortality rate was 16%, three patients (6.8%) were in a persistent vegetative state, two were severely disabled (4.5%), and 11 (25%) suffered mild–moderate disability. All cases demonstrated neurodevelopmental deficits relative to controls. Multiple or large infarcts were prognostic of poor outcome.

**Interpretation:**

Neurological and neurodevelopmental deficits are common after paediatric TBM with hydrocephalus, and appear to be related to ongoing cerebral ischaemia and consequent infarction. The impact of TBM on these children is multidimensional and presents short‐ and long‐term challenges.

## What this paper adds

- Detailed prospective characterization of a paediatric tuberculous meningitis (TBM) cohort.
- Assessment of neurodevelopmental deficit following TBM.
- Assessment of clinical outcome with active management of intracranial pressure.
- Discussion of the acute and long‐term impact of childhood TBM.

This article is commented on by Mouchet on pages 428–429 of this issue.

**Abbreviations**
CSF: Cerebrospinal fluid
GCS: Glasgow Coma Score
GMDS‐ER: Griffiths Mental Development Scales – Extended Version
ICP: Intracranial pressure
MRC: British Medical Research Council
PCPS: Paediatric Cerebral Performance Category Scale
TBM: Tuberculous meningitis

Tuberculous meningitis (TBM) is the most severe form of extra‐pulmonary tuberculosis in children and leads to death or severe disability in half of those affected.^1^, ^2^, ^3^ Contributing factors to poor outcome include delayed presentation and treatment initiation, severity of disease, and hydrocephalus.^1^, ^2^, ^4^, ^5^, ^6^, ^7^ Hydrocephalus occurs in 80% to 90% of patients^1^, ^8^, ^9^ and is more common when there is extensive exudate in the basal cisterns, which increases the risk and severity of vasculitis. Moreover, consequent raised intracranial pressure (ICP) increases the risk of ischaemia because it further reduces the already compromised cerebral perfusion pressure. The reported morbidity after TBM is high, with only 16% to 20% of children returning to their baseline.^1^, ^10^ Most patients have residual neurological deficit including visual impairment,^1^, ^2^ motor deficits,^1^, ^2^ and hearing loss.^1^ Cognitive, behavioural, and developmental morbidities are common and associated with cerebral infarcts post‐TBM;^11^, ^12^, ^13^ however, data on the impact of this disease on neurodevelopment are sparse. This paper examines patients treated for TBM and hydrocephalus at Red Cross War Memorial Children's Hospital (RCWMCH) in terms of admission and clinical characteristics, laboratory and radiological findings, and clinical and neurodevelopmental outcome.

## Method

### Participants

The cohort was selected as part of a prospective study on the temporal profile of biomarkers in TBM conducted between October 2010 and August 2013. Cases included all children treated for definite or probable TBM with associated hydrocephalus to allow serial sampling of cerebrospinal fluid (CSF) for biomarker analysis. Together with the paediatric wards these patients were treated by the neurosurgical team who manage all patients with TBM with hydrocephalus, approximately 20 patients per year. The diagnosis was evaluated according to criteria from a consensus statement^14^ whereby definite cases included patients in whom CSF was positive for tuberculosis culture or acid‐fast bacilli, and the diagnosis of probable TBM was based on a combination of clinical, bacteriological, and radiological criteria. All patients had radiological evidence of hydrocephalus on their admission computed tomography (CT) brain scan (dilated ventricles with or without fluid shift and loss of sulcal markings; mild – visible temporal horns, rounding of the third ventricle; moderate – all ventricles dilated, no transependymal fluid shift; or severe – dilated ventricles, fluid shift, and loss of sulcal markings). Exclusion criteria for the biomarker study included the absence of hydrocephalus, commencement of anti‐tuberculosis treatment for greater than 72 hours before CSF sampling, and treatment of hydrocephalus before diagnosis of TBM. For completeness, basic data from the excluded patient group were also collected.

Data on demographics, medical history, presenting symptoms, and signs were collected. Tuberculous meningitis severity was determined using the refined British Medical Research Council (MRC) criteria^15^ on admission and after 1 week: Stage I – Glasgow Coma Score (GCS) 15 without focal neurological signs; Stage IIa – GCS 15 with neurological deficit/GCS13–14 with/without neurological deficit, Stage IIb – GCS 10 to 12 with/without focal neurological deficit; and Stage III – GCS less than 10 with/without neurological deficits. Preverbal children with a GCS out of 11 had four units added to provide a GCS out of 15 for MRC staging. Laboratory data from CSF included chemistry, white cell count (WCC), tuberculosis diagnostics (culture, polymerase chain reaction, and Gene Xpert [Cepheid] where possible), and drug sensitivity. Human immunodeficiency virus (HIV) reactivity was recorded. Imaging included chest x‐rays, admission brain CT, and brain magnetic resonance imaging (MRI) 3 weeks after treatment initiation.

All patients were treated with the standard four‐drug regimen of rifampicin (20mg/kg), isoniazid (20mg/kg), pyrazinamide (40mg/kg), and ethionamide (20mg/kg) for 2 months followed by 4 to 6 months' continuation of rifampicin and isoniazid, with adjunctive prednisone (2mg/kg/d) daily for the first 3 weeks.^10^ The management of hydrocephalus has been previously described.^16^, ^17^ Briefly, patients underwent an air encephalogram^17^, ^18^ to establish whether hydrocephalus was communicating. If the hydrocephalus was deemed severe, or the patient presented with a low GCS (<8), temporary external ventricular drainage (EVD) was preferred to normalize the ICP in the acute setting. Non‐communicating hydrocephalus was treated with a ventriculoperitoneal shunt (VPS) or an endoscopic third ventriculostomy (ETV). Patients with communicating hydrocephalus or who had an ETV were medically treated with acetazolamide and furosemide in conjunction with serial lumbar punctures for 3 weeks until the ICP had normalized^19^ according to local protocol.^16^ If ICP did not normalize and there were signs of clinical or radiological deterioration, a VPS was inserted.

Outcome was assessed at 6 months post‐diagnosis. Clinical outcome was assessed by two senior neurosurgeons according to the Paediatric Cerebral Performance Category Scale (PCPS).^20^ Outcome scores were dichotomized as 1 to 3 (good outcome: normal function to moderate disability) and 4 to 6 (poor outcome: severe disability or death). Associations between patient, admission, laboratory, and radiology characteristics, and poor outcome were analysed using the chi‐squared, Fisher's exact, or Mann–Whitney *U* tests.

Neurodevelopmental outcome was assessed using the Griffiths Mental Development Scales – Extended Version (GMDS‐ER), the internationally accepted criterion‐standard test of development in children 0 to 8 years, which has been validated in the South African context with English‐, Afrikaans‐, and Xhosa‐speaking children^21^, ^22^ and which has been used to assess neurodevelopment in South African paediatric patients with TBM in two earlier studies.^11^, ^23^ It measures a global developmental quotient made up of six sub‐scales including locomotor (tests physical development including the ability to sit, walk, run, jump, balance, and master stairs), personal‐social (assesses personal and social development including the ability to respond socially to interactions, independence in eating, dressing, and bathroom activities, and knowledge of personal details), hearing and language (examines the growth and development of language through tasks which test vocabulary, sophistication of speech and language expression, as well as comprehension), eye and hand function (assesses the handwork and visual ability of the child with tasks involving building blocks, drawing, and manipulation of objects), performance (tests skill in manipulation, speed of working, precision, pattern recognition, model construction, and pattern‐making), and practical reasoning (for children >2y – focuses on numerical reasoning and simple problem‐solving with tasks involving counting, memory, and reasoning). The general quotient was calculated as an overall score combining all the sub‐scales. Both the Baby scale (0–2y) and 3 to 8 years' test versions were used. Patients were assessed for hearing difficulties, and were assessed in their native language (English, Afrikaans, or Xhosa) with the assistance of an interpreter trained in the GMDS‐ER.

Since RCWMCH patients with tuberculosis often come from impoverished backgrounds it is likely that pre‐morbidly these patients would perform poorly on neurodevelopmental tests designed in a different socio‐economic setting.^24^, ^25^ To isolate the association between development and TBM specifically, we included a control group of children from similar socio‐cultural circumstances (similar neighbourhoods, language, and race groups) who did not have a history of developmental delay, tuberculosis, or other central nervous system disease. First, TBM case and control results were compared to the GMDS‐ER norms in terms of age‐equivalents, and the distribution of scores above and below the normative age‐equivalent was calculated. Next, the median raw score for each sub‐scale and the general quotient were calculated for the control group. This was done in age bands of 0 to 24, 25 to 48, and 49 to 96 months to accommodate for age‐related function. The raw scores per sub‐scale and for the general quotient of the patients with TBM were compared to these control medians; if the raw score fell below the control median the case was defined as having neurodevelopmental deficit. The median was selected as the threshold for comparison because the distribution of scores below the control median most closely matched the distribution of scores below the age‐equivalents of the GMDS‐ER norms.

The relationship between clinical and neurodevelopmental outcomes was analysed. As the GMDS‐ER testing was limited to children physically and cognitively able to participate, clinical outcome was dichotomized as normal (PCPS 1) or mild‐moderate disability (PCPS 2–3), and compared to the patients' neurodevelopmental score (deficit or no deficit) for the general quotient and each sub‐scale using the chi‐squared or Fisher's exact test. Ethics approval was obtained from the Human Research Ethics Board of the University of Cape Town. Patients' parents provided informed consent for study participation.

### Statistical analysis

All analyses were conducted using stata (StataCorp. 2013. Stata Statistical Software: Release 13. College Station, TX: StataCorp LP). Data are presented as median (range or interquartile range) because of the non‐parametric distribution. The significance level was set at 0.05.

## Results

**Figure 1:**
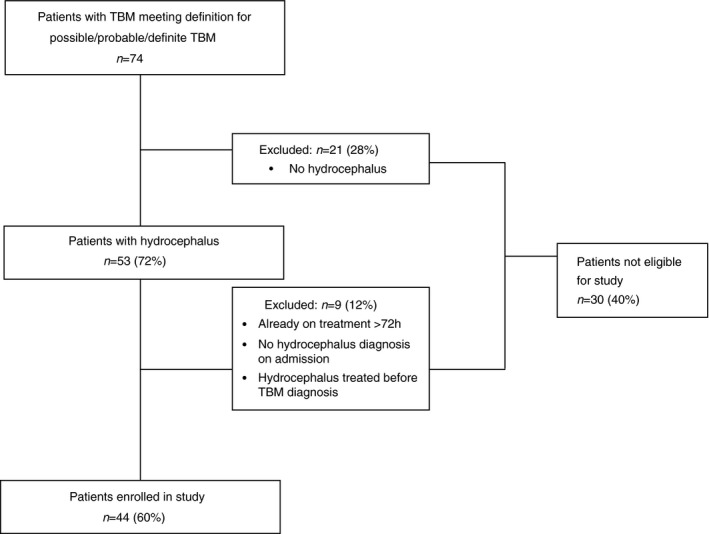
Figure demonstrates the process by which patients eligible for inclusion in the biomarker study were identified from among all the admissions for tuberculous meningitis (TBM) at Red Cross War Memorial Children's Hospital. Patients were referred from primary or secondary health services or presented directly.

Forty‐four patients were included in this study (Fig. 1). The median age for cases was 3 years 3 months (range 3mo–13y[SD 3y 5mo]) and males were more commonly affected (*n*=28). HIV status was tested in 43 patients (97%) and was positive in two children (5%). The median duration of symptoms relating to the TBM infection was 7.5 days. Symptoms of pulmonary tuberculosis were not commonly reported, but weight loss and failure to thrive were noted in half the cohort. Ninety per cent of patients presented with MRC Stage II or III TBM. By 1 week after admission clinical staging had improved in 21 (47.7%) patients and worsened in nine (20.5%). The most commonly recorded signs and symptoms were an altered level of consciousness (*n*=40, 90.1%), meningism (*n*=34, 77.2%), fever (*n*=30, 68.2%), and loss of appetite (*n*=27, 61.4%). Focal neurological signs included unilaterally non‐reactive pupils (*n*=6, 13.6%), other cranial nerve palsies (*n*=10, 22.7%), limb paresis (*n*=12, 27.3%), and aphasia (*n*=8, 18.2%). Demographic and admission characteristics and their association with poor clinical outcome are outlined in Table 1.

**Table 1:** Admission demographic and clinical characteristics

Characteristic	Value		Association with poor outcome, *p*	
Demographic characteristics				
Age, y	3.3 (0.3–13.1) y		0.96	
0–2	19 (43.2)			
3–5	18 (40.9)			
>5	7 (15.9)			
Sex				
Male	28 (63.6)		0.1	
Female	16 (36.4)			
Admission characteristics				
MRC staging	Admission	Week 1	0.01^e^	<0.001^e^
1	4 (9)	18 (40.9)		
2a	17 (38.6)	8 (18.2)		
2b	14 (31.8)	5 (11.4)		
3	9 (20.5)	11 (25)		
Dead	0	2 (4.5)		
Symptom duration	7.5 (1–90) d		0.04^e^	
Weight loss/failure to thrive	22 (50)		0.74	
Night sweats	4 (9)		0.56	
Cough>2wks	8 (18.2)		0.08	
Vomiting	24 (54.5)		0.1	
Lethargy and sleepiness	36 (81.8)		0.66	
Irritability	11 (25)		0.02^e^	
Headache (*n*=37)^c^	15 (40.1)		0.02^e^	
Loss of appetite	27 (61.4)		0.49	
Seizures	14 (31.2)		0.11	
Focal neurological signs^a^	21 (47.7)		0.32	
Altered level of consciousness	40 (90.1)		0.56	
Meningism	34 (77.2)		0.1	
Fever	30 (68.2)		0.52	
Bulging fontanelle (*n*=9)^b^	5 (55.6)			
Papilloedema	9 (20.1)		0.68	
Recent TB contact	21 (47.7)			
Vaccination (*n*=33)^d^	26 (78.8)			
TST (*n*=29)	20 (69)			
HIV infection (*n*=43)	2 (4.7)		0.02^e^	
Diagnostics				
CSF TB culture (*n*=39)			0.56	
positive	21 (53.8)			
negative	18 (46.2)			

^a^Focal neurological signs includes pupillary response, paresis, cranial nerve palsies, and aphasia. ^b^For children with open fontanelles. ^c^Pre‐verbal children under the age of 1 year 6 months excluded. ^d^Parent or guardian not present or Road to Health Card missing. ^e^Statistically significant result. Values reported as median and range or number (percentage). Outcome is dichotomized (good and poor) at 6 months, the association with poor outcome is reported. All outcome analyses were conducted using a chi‐squared or Fisher's exact test, except a Mann–Whitney *U* test was used to test the outcome association for age and symptom duration. TB, tuberculosis; TST, tuberculin skin test; HIV, human immunodeficiency virus; CSF, cerebrospinal fluid.

Thirty‐nine patients had CSF sent for culture. In some cases tuberculosis diagnostics were not requested in error (*n*=4) or the CSF volume was insufficient (*n*=1). Tuberculous meningitis was confirmed in 22 of the 39 patients (56.4%) with positive culture or smear; none had drug resistance. One patient had disseminated tuberculosis confirmed on abdominal imaging, three patients had positive tuberculosis culture on tracheal aspirate, one on gastric wash, and one on sputum. Admission lumbar CSF chemistry (Table 2) demonstrated elevated protein and WCC greater than 10/cu mm in 95% of patients, low glucose in 79% of patients, and low chloride in 92%. Eight patients (21%) had normal admission lumbar CSF glucose; however, two among them developed abnormally low glucose in their second samples. Only two patients (5.3%) had normal admission lumbar CSF protein values and a total WCC less than 10/cu mm; however, these were all elevated by the second sample. Lymphocytic predominance greater than 50% was common (84%). Three patients (8.3%) had initially normal lumbar CSF chloride values, which decreased below the reference range in the first 10 days of hospitalization. Perturbations in CSF chemistry and elevated lymphocyte count were not significantly associated with poor outcome (*p*>0.05). Elevated polymorphonuclear cells in the lumbar CSF trended towards an association with better outcome at 6 months (*p*=0.06).

**Table 2:** Admission cerebrospinal fluid chemistry and cell counts

CSF chemistry and white cell count	Patients with TBM (*n*=38)	Association with poor outcome, *p*
Glucose (mmol/L)	1.6 (0.3–4.8)	0.4
Normal (2.3–3.9)	8 (21)	
Abnormal (<2.3)	30 (78.9)	
Chloride (mmol/L)^a^	106.5 (93.0–131.0)	0.78
Normal (120–130)	3 (8.3)	
Abnormal (<120)	33 (91.7)	
Protein g/L	1.98 (0.34–40.88)	0.2
Normal (0.2–0.8/0.15–0.45)	2 (5.3)	
Abnormal (>0.45/>0.8)^b^	36 (94.7)	
Polymorphonuclear	18.5 (0–280.0)	0.06^c^
Cells (/cu mm)		
Lymphocytes (/cu mm)	144 (6.0–715.0)	0.25
Total white cell count	169 (0–901.0)	
Normal (<10)	2 (5.3)	
Abnormal (>10)	36 (94.7)	
Lymphocyte predominance		
>50%	32 (84)	
>90%	14 (36.8)	
CSF/serum albumin ratio	29.68 (0.3–588.6)	0.12
Normal	1 (3.1)	
Abnormal	31 (97)	

^a^Admission chloride values were only available for 36 patients. ^b^Protein reference ranges are age‐dependent: >1y and <1y respectively, an association with poor outcome using Mann–Whitney *U* test is shown. ^c^Statistically significant. Data presented as median (range), *n (%)*. CSF, cerebrospinal fluid. TBM, tuberculous meningitis.

Hydrocephalus was communicating in 34 patients (79.1%), non‐communicating in three patients (7%), and uncertain in seven patients. The median opening pressure on admission LP was 24 cmH_2_O (1–51 cmH_2_O). Temporary EVDs were used as an initial intervention in 31 patients (70.5%). Medical treatment was successful in 57.1% of patients with communicating hydrocephalus. Overall, 25 patients (56.8%) had a VPS inserted as part of early hydrocephalus treatment or after failed medical treatment.

Admission head CT showed hydrocephalus in all patients (*n*=44), focal or diffuse enhancement in 95.5% (*n*=41), tuberculomas in 6.8% (*n*=3), and infarcts in 16% (*n*=7). At the 3‐week MRI, 64% (*n*=25) demonstrated infarction.

### Patient outcome

Mortality by 6 months was 16% (*n*=7). Four patients died within the first 10 days after admission (median 4d), two patients died after 7 weeks, and one patient died after 6 months. Mortality was higher among females (*p*=0.05). British MRC staging at 1 week was predictive of mortality (*p*<0.001); more deaths occurred in severe categories IIb and III; MRC staging at admission approached significance in association with mortality (*p*=0.06). Outcome data are presented in Table 3. Almost half of the cohort (*n*=21) made a good clinical recovery with no neurological morbidity, despite 67% of them presenting with a decreased level of consciousness; 16 patients (36.6%) suffered some form of disability: motor deficit (*n*=11), visual deficit (*n*=4), hearing deficit (*n*=2), cranial nerve palsy (*n*=1); and three patients (6.8%) were considered vegetative. Admission characteristics which were significantly predictive of poor PCPS included: MRC staging on admission (*p*=0.01), and at week 1 (*p*<0.001), and a shorter duration of symptoms (*p*=0.05). Bilateral (*p*<0.001) and large infarcts (*p*<0.001) involving multiple vascular territories (*p*<0.001*)* were predictive of poor outcome. Multi‐variate analysis did not identify any independently associated variables.

**Table 3:** Clinical outcome at 6 months

Stage	MRC staging on admission				Total
	I	IIa	IIb	III	*n*=44
Full recovery (PCPS 1)	3 (6.8)	12 (27.3)	6 (13.6)	0	21 (47.7)
Mild disability (PCPS 2)	1 (2.3)	2 (4.5)	3 (6.8)	2 (4.5)	8 (18.2)
Moderate disability (PCPS 3)	0	1 (2.3)	1 (2.3)	1 (2.3)	3 (6.8)
Severe disability (PCPS 4)	0	0	2 (4.5)	0	2 (4.5)
Vegetative (PCPS 5)	0	1 (2.3)	0	2(4.5)	3 (6.8)
Died (PCPS 6)	0	1 (2.3)	2 (4.5)	4 (9.1)	7 (15.9)

Stage	MRC staging at week 1				Total
	I	IIa	IIb	III	*n*=42
Full recovery (PCPS 1)	15 (35.7)	5 (11.9)	1 (2.4)	0	21 (50)
Mild disability (PCPS 2)	3 (7.1)	2 (4.8)	1 (2.4	2 (4.8)	8 (19)
Moderate disability (PCPS 3)	0	1 (2.4)	2 (4.8)	0	3 (7.1)
Severe disability (PCPS 4)	0	0	0	2 (4.8)	2 (4.8)
Vegetative (PCPS 5)				3 (7.1)	3 (7.1)
Died (PCPS 6)	0	0	1 (2.4)	4 (9.5)	5 (11.9)

This table presents clinical outcome data according to British Medical Research Council (MRC) staging on admission and at week 1. Data are presented as number (per cent). Data for Week 1 are presented out of 42 patients, two patients had already died by week 1. PCPS, Paediatric Cerebral Performance Category Scale.

### Neurodevelopmental outcome

Twenty‐six patients with TBM (*n*=18 males) with a median age of 3 years (range 1–6y 3mo) underwent neurodevelopmental assessment. Patients older than the age limit of the test (>8y, *n*=5), with severe clinical disability (*n*=5), who had died (*n*=7), and who had documented pre‐morbid neurodevelopmental delay (*n*=1) were not evaluated. All auditory assessments were normal; one patient had visual deficits. Most of the mothers had some high‐school education (85%), and 12% had completed school. The control group comprised 25 children (*n*=15 males) who were matched as best as possible on age, sex, language, social background, and area of residence. They had a median age of 3 years 5 months (range 10mo–6y 10mo). Their mothers had some high‐school education (36%) or had completed school (64%).

In comparison to GMDS‐ER norms, the control group performed at an age‐appropriate level for the Locomotor and Personal‐Social subscales, but the majority of controls performed below their age equivalent on the Eye‐Hand coordination, Performance, Language, and Reasoning subscales. The control general quotient scores fell within the first standard deviation of the equivalent normative general quotient for age. Most of the patients with TBM performed at a level below their age equivalent on all subscales and scored a median of three standard deviations below the normative general quotient for age, as detailed in Table 4. In comparison to control group median values, the number of patients with TBM who scored below the median on each sub‐scale and the general quotient was as follows: 22 (84.6%) on the Locomotor sub‐scale; 23 (88.5%) on Personal‐Social; 18 (69.2%) on Language; 19 (73.1%) on Eye‐Hand co‐ordination; 20 (76.9%) on Performance; 11 (78.6% – only 14 patients were old enough to be tested on this scale, >2y) on the Reasoning sub‐scale; and 21 (80.8%) on the general quotient. No statistically significant association was demonstrated between the clinical and neurodevelopmental scores.

**Table 4:** Control and case scores in relation to Griffiths Mental Development Scales – Extended Version normative age equivalents

	Normal controls (%)	Patients with TBM (%)
Locomotor		
<age equiv	2 (8)	24 (92.3)
≥age equiv	23 (92)	2 (7.7)
Personal/social		
<age equiv	2 (8)	20 (76.9)
≥age equiv	23 (92)	6 (23.1)
Language		
<age equiv	19 (76)	24 (92.3)
≥age equiv	6 (24)	2 (7.7)
Eye‐hand coordination		
<age equiv	17 (68)	26 (100)
≥age equiv	8 (32)	0 (0)
Performance		
<age equiv	14 (56)	25 (96.2)
≥age equiv	11 (44)	1 (3.8)
Reasoning^a^		
<age equiv	13 (76.5)	14 (100)
≥age equiv	4 (23.5)	0 (0)
GQ		
<age equiv	12 (48)	26 (100)
≥age equiv	13 (52)	0 (0)

^a^Only children >2y could be assessed on this sub‐scale. Data reported as number (per cent). TBM, tuberculous meningitis; GQ, general quotient; equiv=equivalent.

### Data for included and excluded patients with TBM

Seventy‐four patients met the consensus criteria for definite or probable TBM during the study period (Fig. 1). Hydrocephalus was diagnosed in 53 patients (71.6%); 44 (83%) patients were enrolled in the biomarker study; and nine (17%) were excluded according to the biomarker study criteria. The median age for the 74 patients was 3 years 9 months (range 3mo–13y) and 42 (56.7%) were males. Cerebrospinal fluid culture was positive in 31 (42%) patients and two had mono‐drug resistance. The HIV positivity rate was 8% (*n*=6) and the mortality rate was 13.5% (*n*=10).

## Discussion

Among the study patients treated at RCWMCH for definite or probable TBM and hydrocephalus, almost 50% demonstrated an improvement in their MRC staging after 1 week in hospital, likely as a consequence of treating reversible factors such as increased ICP, seizures, and hyponatraemia. The CSF tuberculosis culture positivity yield of 53% for the study population was a marked improvement on historical yields at our institution (approximately 30%), was higher than in the non‐hydrocephalus group (33%), and higher than the 11.7% culture yield reported over 20 years recently.^1^ This may be because of more samples and the larger volumes of CSF sent for microbiology.^26^ Laboratory findings demonstrated typically low glucose, high protein, and lymphocytic predominance. In the few samples in which this was not initially the case, most of the subsequent samples revealed these patterns, highlighting the value of repeated CSF testing in presumptive TBM diagnosis.

Despite rigorous treatment of hydrocephalus and raised ICP, death and disability were common. This was likely because of ongoing ischaemia as demonstrated by infarcts in more than two‐thirds of patients. However, our results may overestimate poor outcome in childhood TBM, given that the study population was selected for more severe disease and because of referral patterns to a specialist hospital with advanced critical care facilities. Most patients who died or suffered severe disability deteriorated within the first week post‐admission, emphasising the importance of early diagnosis and demonstrating the acute progression of TBM disease in children who suffer poor outcomes. On the other hand, patients who responded to treatment did well despite initial depressed neurological function, and almost half the cohort made a good clinical recovery and disabilities were often mild, suggesting that good clinical outcomes are possible with prompt appropriate treatment. Therefore, there is an imperative for research that examines novel biomarkers or diagnostic tests to enable quicker diagnosis of this condition so that patients may be treated before the severity of the disease progresses. In the absence of a currently available sensitive and specific point‐of‐care diagnostic test for TBM, our data show that CSF chemistry and haematology markers in combination with imaging findings of basal enhancement and hydrocephalus are valuable tools for clinicians to make a presumptive diagnosis and commence treatment promptly so as to increase the chance of a good response to treatment and outcome. However, 80% of the patients who underwent neurodevelopmental follow‐up demonstrated deficits in locomotion, language, co‐ordination, personal, social, and executive function, despite many having made a good clinical outcome. These deficits are compounded by the fact that most of these children stem from impoverished homes with limited child‐caring options, and from communities with a shortage of schools able to provide adequate support.

The associations between admission characteristics and clinical outcome were weak. It was an unexpected finding that a shorter duration of symptoms was associated with poor outcome. This may reflect the aggressive nature of the disease in the patients who died, as opposed to a low‐level chronic disease process in children who survived. There were more deaths in females; however, with the small number of deaths (*n*=7) this may be a chance finding. Higher polymorphonuclear cell counts appeared to be associated with better outcome, and there are data to suggest that neutrophils contribute to the early defence against mycobacterium tuberculosis and that the recruitment of these cells to the site of disease improves outcome.^27^ It is possible, therefore, that patients who had higher neutrophil counts were admitted at an earlier stage of TBM disease, and were consequently more likely to have a good outcome.

Prolonged hospitalization may also have affected patient neurodevelopment: children were fed, dressed, and washed by nursing staff, and consequently did not develop independence in these tasks; they were spoken to in a mixture of languages; they were not exposed to environments external to the hospital ward and playground; teaching was limited by poor resources; and there was prolonged maternal/family separation with consequent lack of input. The impact of TBM on development is, therefore, multidimensional, comprising the organic injury and the environmental, social, and educational limitations imposed by prolonged treatment.

The absence of an association between neurodevelopmental and clinical outcome may be due to differences in the outcome being assessed. Clinical outcome focused on neurological deficits discernible on physical examination. The GMDS‐ER assessed a broad range of functional categories and examined whether an activity could be performed, as well as the quality with which it was performed: therefore, it detected both gross and subtle deficits. Additionally, clinical outcome is determined largely by pathology, whereas neurodevelopmental outcome is influenced by pathology in combination with social, educational, and environmental factors. Behavioural questionnaires were not conducted with parents as most of these patients were cared for at a long‐stay tuberculosis hospital and, therefore, parents may have been unaware of behavioural problems.

### Limitations

Admission data may have been limited by variable qualities of history‐taking and the accuracy of parental reporting. Placing outcome of this hydrocephalus study group in the context of overall TBM outcomes at RCWMCH was limited, because non‐hydrocephalus patients are followed up at other hospitals. For the neurodevelopmental outcome it was not possible to exactly match cases and controls, and several of the control children's mothers were more educated. However, the average age of the children with mothers who had tertiary education was 13.5 months. Molteno et al.^24^ study found that child development was similar across all socio‐economic status groups until approximately 12 months of age. Therefore, some of the discrepancy between maternal levels of education may have been mitigated. Establishing an appropriate control group is challenging given the number of variables of potential relevance, and although the GMDS‐ER has been used for several local TBM studies they have not included a control group.^11^, ^23^

## Conclusion

Childhood TBM can be devastating in terms of mortality and disability as a result of injury to the brain, and the developmental implications are further exacerbated by long‐term hospitalisation within a resource‐limited health system. Physical and cognitive deficits pose serious challenges to patients, their families, and communities who do not have access to adequate rehabilitative and educational resources, and amplify the long‐term impact of this disease. However, good outcomes are possible in patients who respond well to prompt appropriate treatment.
